# A phase 1 open-label pilot study of low-dose interleukine-2 immunotherapy in patients with Alzheimer’s disease

**DOI:** 10.1186/s40035-023-00387-5

**Published:** 2023-11-16

**Authors:** Alireza Faridar, Abdulmunaim M. Eid, Aaron D. Thome, Weihua Zhao, David R. Beers, Maria B. Pascual, Mohammad O. Nakawah, Gustavo C. Roman, Charles S. Davis, Michael Grundman, Joseph C. Masdeu, Stanley H. Appel

**Affiliations:** 1 Stanley H. Appel Department of Neurology, Houston Methodist Research Institute, 6565 Fannin Street, Suite P3-201, Houston, TX USA; 2 CSD Biostatistics, Inc., Oro Valley, AZ USA; 3 Global R&D Partners, LLC, San Diego, CA USA; 4 University of California San Diego, San Diego, CA USA

## Abstract

ClinicalTrials.gov Identifier: NCT05821153, Registered April 20 2023, Retrospectively registered, https://classic.clinicaltrials.gov/ct2/show/NCT05821153

**Supplementary Information:**

The online version contains supplementary material available at 10.1186/s40035-023-00387-5.

Regulatory T cells (Tregs) constitute a subset of T cells that play a protective role by suppressing inflammation [1]. We previously documented that the Treg immunomodulatory mechanisms are compromised in AD patients [2], resulting in an activation of peripheral monocytes, associated with upregulation of inflammatory mediators [3]. Preclinical studies have suggested variable effects of Treg modification on the neurodegenerative process. While some studies propose that the Treg population might obstruct a selective gateway for immune cell trafficking to the CNS, thereby compromising reparative immune responses [4, 5], an increasing number of preclinical studies, including ours, indicate that systemic Treg expansion through interleukine-2 (IL-2) administration or ex vivo expanded Treg administration effectively modulates neuroinflammation and alleviates AD pathology [6, 7]. IL-2, originally described as the main T-cell growth factor, has been used in standard high doses for activation of cytotoxic T cells and NK cells [8]. However, at low doses IL-2 will preferentially bind Tregs because of their constitutively expressed high-affinity IL-2 receptor complex [9]. Low-dose IL-2-induced Treg expansion has been shown to be safe in inflammatory diseases and has demonstrated preliminary indications of biological and clinical efficacy [10]. However, IL-2-induced expansion of Tregs has not been evaluated in an AD clinical setting.

This study is an open-label, phase-1 trial, designed to evaluate the safety and feasibility of low-dose IL-2 to expand Tregs in AD individuals (*n* = 8). Participants with elevated brain amyloid, as demonstrated either through cerebrospinal fluid or by amyloid positron emission tomography scan at screening, were eligible. The main inclusion criteria were age between 60 and 86 and Global Clinical Dementia Rating scale of 1. Baseline characteristics of the recruited individuals are presented in Additional file 1: Table S1. Written informed consent was obtained following ethics approval from the Institutional Review Board. Within 2 weeks after the screening visit, patients received a daily fixed dose of subcutaneous recombinant human IL-2 (Aldesleukin, Clinigen) (1 × 10^6^ units/dose) injection for 5 consecutive days. This 5-day cycle was repeated three more times, on days 30–35, 60–65 and 90–95. After the last treatment cycle, all patients were followed up for safety monitoring on days 120 and 168. Blood samples were obtained serially on day 0 (before IL-2 administration) and day 8 (3 days after the last dose) of each treatment cycle as well as on days 120 and 168. MMSE was administered at baseline and every 30 days during the treatment phase, and then on days 120 and 168. Clinical Dementia Rating Scale Sum of Boxes (CDR-SB) and Alzheimer’s Disease Assessment Scale Cognitive Subscale (ADAS-Cog) were scored at baseline and on days 120 and 168. More details on outcome measures are available in the Additional file 1: methods.

All 8 patients completed the four-month treatment phase as well as the 2-month post-treatment follow-up phase. There were no serious adverse events reported following IL-2 administration. The most common adverse events were injection site irritation/redness (37.5%), mild leukopenia (37.5%), flu-like symptoms (12.5%), dizziness (12.5%) and nausea (12.5%). The percentage and immunophenotype of peripheral immune populations were assessed by flow cytometry. For the secondary endpoint, the CD4^+^FoxP3^+^CD25^high^Treg percentage of total CD4 T cells increased from the baseline value of 4.55% ± 0.70% to 8.68% ± 1.06% on day 98, a difference of 4.13% ± 0.65% (*P* = 0.0004). More generally, the Treg percentage increased to 1.8–2.2 folds after each IL-2 treatment cycle and returned to baseline before the next cycle (Fig. 1a). Whether the IL-2 treatment increased the number and/or function of polyclonal or antigen-specific Treg subsets in AD individuals will require further investigation at the single cell levels. In contrast to Tregs, the percentages of CD4^+^CD25^low^T responders (Tresps) were reduced after IL-2 administration (Additional file 1: Fig. S1a). Like Treg percentage, the CD25 mean fluorescence intensity (MFI) in the Treg population was amplified following IL-2 administration (Fig. 1c). FoxP3 MFI increased only after the first cycle of IL-2 administration (Additional file 1: Fig. S1b). No changes were noted in CD8^+^T cell or CD56^+^ natural killer populations (Additional file 1: Fig. S1c, d). Tregs and Tresps were also co-cultured at a Treg:Tresp ratio of 1:1 or 1/2:1. The Treg suppression of Tresp proliferation, at baseline, was 46.6% and 29.8% at 1:1 and 1/2:1 Treg:Tresp ratios, respectively. The suppressive function of Tregs at both ratios was increased on day 8, day 38, day 60, day 68 and day 98 of IL-2 treatment phase (Fig. 1b). Peripheral monocytes were also isolated from the same blood samples and IL-1β, TNF and IL-6 transcripts were analyzed using RT-PCR. The expression of pro-inflammatory cytokine transcripts was down-regulated in the monocyte population throughout the 4-month IL-2 treatment phase (Fig. 1d). The plasma levels of 45 selected chemokines and cytokines were measured longitudinally using the Olink® Target Cytokine Panel. The level of the pro-inflammatory cytokine IL-15 decreased following each IL-2 treatment cycle and returned toward baseline level before subsequent treatment (Fig. 1e). A decreasing trend or statistically significant attenuation of the plasma levels of macrophage/microglial activation chemokines C–C motif ligand (CCL)-2, CCL4 and CCL11 (Fig. 1f–h) was also noted following IL-2 administration. A similar pattern was observed in the longitudinal analysis of dendritic growth factor, FLT3LG (Fig. 1i). The pro-inflammatory cytokine TNF also trended downward through the IL-2 treatment phase and was significantly reduced on days 30 and 38 of treatment phase (Fig. 1j). No changes were noted in the longitudinal analyses of other measured plasma immune markers. There were improvements in MMSE scores on days 30, 60, and 90 during the IL-2 treatment phase, and the MMSE score returned toward baseline after discontinuation of the treatment (Fig. 1k). A trend toward improvement was observed for CDR-SB on day 120 which was reversed on day 168 (Fig. 1l). The ADAS-Cog scores on days 120 and 168 were comparable to the baseline levels (Fig. 1m).

**Fig. 1:**
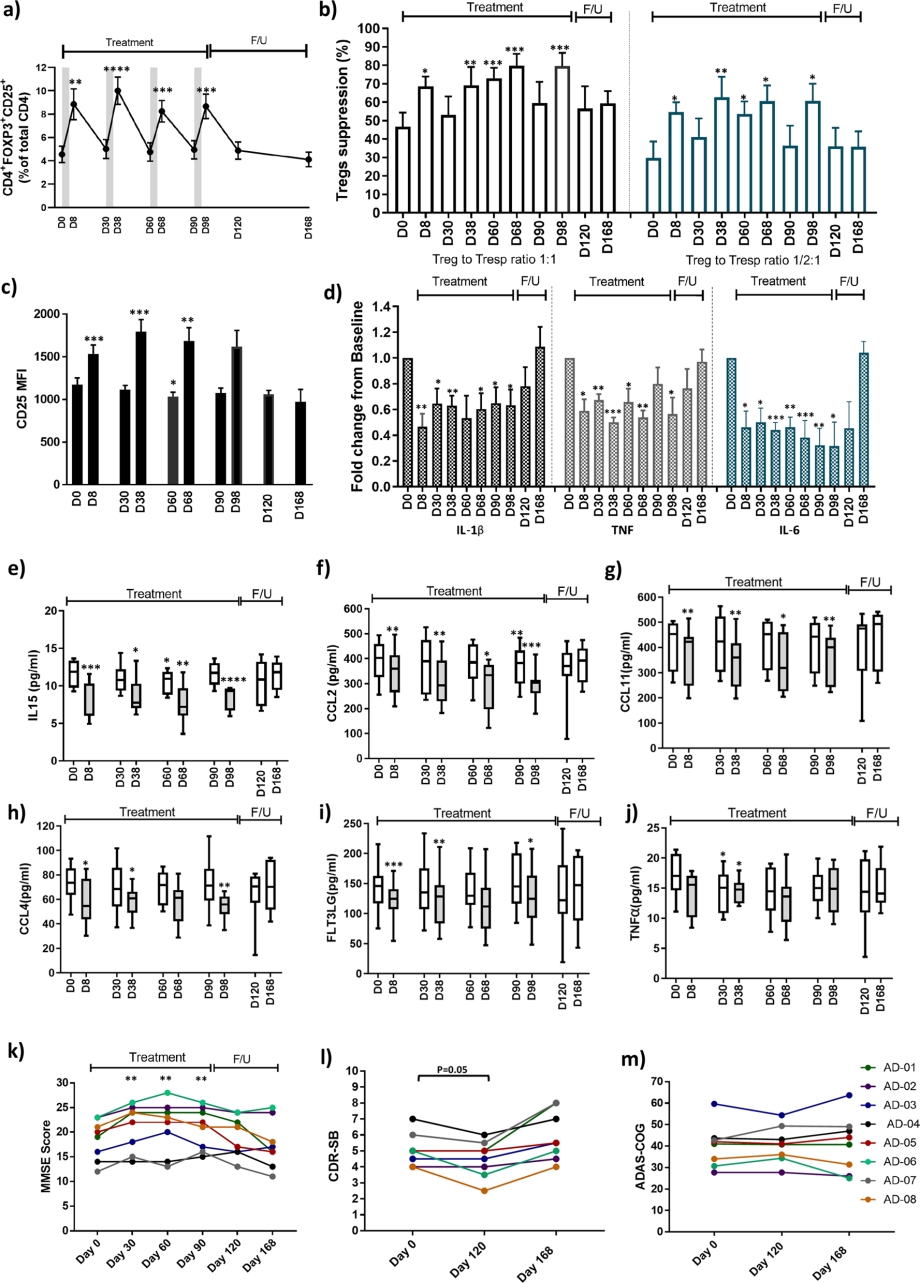
Effect of low-dose IL-2 treatment on immune parameters and cognitive status in AD subjects.** a** Percentage of FOXP3^+^CD25^high^ CD4 T cells (i.e.,Tregs) was amplified 3 days following each IL-2 treatment cycle and returned to baseline before the next cycle. **b** The suppressive function of Tregs at both 1:1 and 1/2:1 Treg:Tresp ratios improved throughout the 4-month IL-2 treatment phase and reached higher levels on days 8, 38, 60, 68 and 98 of IL-2 treatment phase, compared to the baseline levels (D0). After completion of IL-2 treatment phase, the suppressive function of Tregs trended toward baseline levels on days 120 and 168. **c** CD25 MFI in Treg population was increased following IL-2 administration on days 8, 38 and 68. **d** Transcript expressions of IL-1β, TNF and IL-6 in monocyte population were downregulated in the IL-2 treatment phase. A decreasing trend or statistically significant attenuation of plasma IL-15 **(e)**, CCL2 **(f)**, CCL11 **(g)**, CCL4 **(h)**, FLT3LG **(i)** and TNF **(j)** was noted following IL-2 administration. MMSE scores were improved through the IL-2 treatment phase and returned toward baseline after discontinuation of the treatment **(k)**. A trend toward improved CDR-SB was observed on day 120, compared to baseline **(l)**. The changes in the average of ADAS-Cog scores were not significant through this 24-week study **(m)**. Numbers shown represent the mean ± SE. Mean change from baseline was evaluated with paired *t*-tests. *P < 0.05, **P < 0.01, ***P < 0.001, and ****P < 0.0001. Treg: Regulatory T cells, IL-2: Interleukin-2, MMSE: Mini-Mental State Examination, CDR-SB: Clinical Dementia Rating Scale Sum of Boxes, ADAS-Cog: Alzheimer’s Disease Assessment Scale Cognitive Subscale, CCL: C–C motif ligand

The recently approved anti-amyloid immunotherapies offer partially effective treatment for a relatively small subset of the vast and expanding AD population [11]. There is a major need to diversify the drug development pipeline in AD clinical setting. Treg expansion strategy has been translated into the clinical setting for neurodegenerative disorders [12]. In the current study, low-dose IL-2 administration safely expanded the Treg population, suppressed peripheral pro-inflammatory monocytes and reduced plasma myeloid activating chemokines in AD subjects. Improvements were observed on some clinical assessments (MMSE) following IL-2 administration; however, interpretation of this finding is limited by the lack of a placebo group, the small sample size, and the short duration of treatment. To confirm and expand on our findings, we are presently conducting a phase 2a double-blinded, randomized, placebo-controlled clinical trial to assess low-dose IL-2 immunotherapy in subjects with AD. In this proof-of-concept trial, the impact of IL-2 immunotherapy on established AD biomarkers, cognitive and functional endpoints are being assessed. Additionally, we will investigate whether the IL-2-induced peripheral Treg expansion will modify neuroinflammation in AD individuals.

### Supplementary Information

**Additional file 1**. Materials and Methods. **Table S1**. Baseline characteristics of the study participants. **Fig. S1.** Effect of low dose IL-2 treatment on peripheral immune parameters

## Data Availability

The datasets supporting the conclusions of this article are included within the article and its additional supplementary file.
