# Supplementary material for: A phase 1 open-label pilot study of low-dose interleukine-2 immunotherapy in patients with Alzheimer’s disease
Source: Transl Neurodegener. 2023 Nov 16;12:54. doi: 10.1186/s40035-023-00387-5 (PMC10652426; doi:10.1186/s40035-023-00387-5)
Supplement: Supplementary file 1 — Additional file 1. Materials and Methods. Table S1. Baseline characteristics of the study participants. Fig. S1. Effect of low dose IL-2 treatment on peripheral immune parameters [file 40035_2023_387_MOESM1_ESM.docx]

**Supplementary Materials and Methods:**

**Safety measures** were monitored throughout the trial by characterization of adverse events, chemistry and hematology laboratory values, vital signs, and physical and neurological examinations. Tolerability was defined as the percentage of participants who completed the treatment period of the trial.

**For flow cytometric analysis**, antibodies against the following molecules and corresponding IgG isotype controls were provided by: CD3 BV650 (BD Biosciences), CD4 APC-H7 (BD Biosciences), CD25 PerCPCy5.5 (BD Biosciences) CD8 BV450 (BD Biosciences), CD16 PE-Cyanine7 (eBioscience) and CD56 APC (eBioscience). For intracellular staining, cells were fixed and permeabilized using the FoxP3/Transcription Factor Staining Buffer Set (eBioscience), and then stained with FoxP3 Alexa Fluor 488 (eBioscience).

**To evaluate changes in Treg immunosuppressive function**, we isolated CD4^+^CD25^high^Tregs and CD4^+^CD25^low^ T responders (Tresp) in a 2-step procedure, using a Treg isolation kit (Miltenyi Biotec). Isolated Tregs and Tresps were co-cultured at 1:1 and 1:1/2 ratio (Tresp:Treg) for 5 days in the presence of a CD3/CD28 T cell stimulation reagent from Miltenyi Biotec. This stimulus is specific to Tresps and does not cause proliferation of Tregs. Proliferation of Tresps was measured via tritium incorporation and calculated as suppression of Tresp proliferation in the presence of Tregs.

**Monocyte transcript analysis:** From the same blood samples, monocytes were negatively selected, using the Human Pan Monocyte Isolation Kit (Miltenyi Biotec). Using Trizol reagent, followed by Direct-zol RNA MiniPrep Kit (Zymo Research), messenger RNA was extracted from the isolated monocyte population. Quantitative PCR experiments were performed using a One-Step RT-PCR kit with SYBR Green and were run on the Bio-Rad iQ5 Multicolor Real-Time PCR Detection Systems. Primers for the study were purchased from BioRad and the relative expression level of each messenger RNA was calculated using the ΔΔCt method with normalization to β-actin and relative to control samples.

**Plasma inflammatory chemokine and cytokine levels** were analyzed with the Olink® Target 48 Cytokine panel (Olink Proteomics, Uppsala, Sweden). This panel uses a multiplex proximity extension assay (PEA) technology to enable measurement of 45 selected cytokines and chemokines simultaneously from small volumes of plasma, through a service provided by the manufacturer.

**To assess cognitive status**, Mini-Mental State Examination (MMSE) was performed at baseline, Days 30, 60, 90, 120 and 168. Two other clinical measures, the Clinical Dementia Rating Scale Sum of Boxes (CDR-SB) and Alzheimer’s Disease Assessment Scale Cognitive Subscale (ADAS-Cog) were assessed at baseline, day 120, and day 168.

**Statistical analysis:** Immune parameters were evaluated by descriptive statistics at each time point and analyzed using two-sided paired t-tests to compare changes from baseline to other assessment timepoints throughout the course of the study period. Change from baseline was calculated as the absolute change except for IL1B, TNF and IL6 where change from baseline was measured as fold change. Change from baseline data are expressed as the mean change ± standard error (SE). The study secondary endpoint of change in Treg percentage from baseline to day 98 was evaluated at the α=0.05 level to determine the overall statistical significance of the study. Changes from baseline on other endpoints were considered nominally significant if the corresponding p-value was <0.05.

**Table S1- Baseline characteristics of the study participants**

|  | **Age** | **Gender** | **MMSE** | **CDR** | **ADAS-Cog** | **PIB- PET** | **Tau-PET** | **AD CSF signature** | **Neurodegeneration in MRI** |
| --- | --- | --- | --- | --- | --- | --- | --- | --- | --- |
| **AD1** | 63 | F | 19 | 5 | 41 | + | + |  | + |
| **AD2** | 65 | F | 23 | 4 | 27.66 | + | + |  | + |
| **AD3** | 69 | F | 16 | 4.5 | 59.66 | + | N/A |  | + |
| **AD4** | 74 | M | 14 | 7 | 43.66 | N/A | N/A | + | + |
| **AD5** | 77 | M | 20 | 5 | 42 | N/A | N/A | + | + |
| **AD6** | 75 | F | 23 | 5 | 30.66 | + | N/A |  | + |
| **AD7** | 70 | M | 12 | 6 | 42.66 | + | N/A |  | + |
| **AD8** | 72 | F | 21 | 4 | 34 | + | N/A |  | + |
| **Mean± SD** | 70.6±4.8 | M/F:3/5 | 19.0±4.3 | 5.1±1.0 | 40.1±9.9 |  |  |  |  |

**Supplementary Figure-1:** **Effect of low dose IL-2 treatment on peripheral immune parameters.**  **a)** The percentage of peripheral CD4^+^CD25^low^ Tresps decreased following IL-2 administration on D8, D38 and D68. **b)** FoxP3 MFI in Treg population was only amplified after the first IL-2 cycles administration on D8. The percentage of peripheral CD3^+^CD8^+^ T cells **(c)** and CD3^-^CD56^+^ natural killer cells **(d)** did not alter following IL2 administration. Grey areas indicate the periods on IL-2 therapy. D= Day. Numbers shown represent the mean ± SE. Mean change from baseline was evaluated with paired t tests. *P*-values are **P* < 0.05, ***P* < 0.01 and ****P* < 0.001.
